# Editorial perspective: Connecting transdiagnostic mechanisms targeted in cognitive behavioral therapies with empirically‐based symptom dimensions

**DOI:** 10.1002/jcv2.70161

**Published:** 2026-08-20

**Authors:** Kasey Stanton, Jack D. Burke, Helena Towne, Isabella Whitesides

**Affiliations:** ^1^ University of Wyoming Laramie Wyoming USA

**Keywords:** cognitive behavioral therapy, dimensional models, third‐wave therapies, transdiagnostic

## Abstract

Various avenues of transdiagnostic research hold promise for improving diagnosis and treatment but remain fragmented in some ways. We provide recommendations for integrating dimensional assessment guided by the Hierarchical Taxonomy of Psychopathology (HiTOP) framework into clinical practice by more effectively connecting the HiTOP with social‐cognitive vulnerability (SCV) mechanisms targeted in cognitive behavioral and third‐wave therapies. Specifically, we present concrete ideas for (a) comprehensively understanding HiTOP symptom dimension‐SCV associations, (b) integrating measure development efforts spanning the HiTOP and SCV literatures, and (c) sharpening terminology for improving construct description and clinical communication. We draw on studies from the 2026 Special Issue of *JCPP Advances on Transdiagnostic Approaches to Child and Adolescent Mental Health* that provide helpful examples illustrating these points, and we use these study examples to demonstrate how HiTOP‐based assessment connects with personalized treatment planning.

## Key Points

**What's Known?**

- Clinical assessment guided by the Hierarchical Taxonomy of Psychopathology (HiTOP) may be more empirically‐based than traditional categorical approaches, and social‐cognitive vulnerabilities (SCVs) are key treatment targets in cognitive behavioral and related therapies.

**What's New?**

- More comprehensively integrating HiTOP and SCV research can sharpen understanding of associations between symptoms and treatment mechanisms, thereby improving the HiTOP's clinical utility.
- Efficient measures are needed to ensure a range of transdiagnostic HiTOP and SCV dimensions can be jointly assessed in research and practice.
- Careful consideration of terminology used in transdiagnostic research can further enhance the utility of dimensional assessment.

**What's Relevant?**

- Integrative models of the HiTOP and SCVs can potentially streamline clinical assessment, while also guiding more personalized treatment planning.

Research on transdiagnostic assessment, mechanisms, and treatment has grown rapidly over the last several decades, as illustrated by the diverse range of methodological approaches featured in this special issue. This editorial provides ideas for bridging two complementary areas of transdiagnostic research focused on (a) social‐cognitive vulnerabilities (SCVs) and (b) classification and symptom assessment guided by the Hierarchical Taxonomy of Psychopathology (HiTOP; see Carrington et al. (2026) from this special issue for an example study informing HiTOP structure). Many cognitive behavioral and related third‐wave treatments target SCVs (e.g., experiential avoidance, unhelpful thinking styles), which are conceptualized as factors contributing to the development and maintenance of an array of symptoms (Gámez et al., 2011). Next, the HiTOP offers a hierarchical, dimensional alternative to traditional models of classification and diagnosis. Key HiTOP spectra include internalizing, disinhibited externalizing, and other spectra, and from a HiTOP perspective, symptoms can be validly classified and assessed across levels of abstraction (e.g., as indicators of broad spectra or more specific subspectra). However, one persistent criticism of the HiTOP is that it does not adequately account for symptom development, maintenance, and course given that it is a largely descriptive framework, similar to traditional categorical models. Thus, research focused on more fully connecting the HiTOP with SCVs across the lifespan may enhance its clinical utility and improve its uptake, by sharpening understanding of how HiTOP symptom dimensions connect with core mechanisms targeted in cognitive behavioral treatments.

We describe three avenues for more fully integrating HiTOP and SCV transdiagnostic research, drawing on articles from the 2026 Special Issue of *JCPP Advances on Transdiagnostic Approaches to Child and Adolescent Mental Health*. First, we describe the value of assessing symptoms across multiple HiTOP spectra and SCVs to optimize symptom assessment‐mechanism research, which in turn, can enhance treatment planning efforts. Second, we provide ideas for developing efficient, transdiagnostic measures that adequately differentiate SCVs from symptom experiences. Third, we discuss how efforts focused on integrating lived experience perspectives can help sharpen terminology used in the two areas of research reviewed previously while also improving clinical communication in practice.

## MAPPING DIMENSIONAL SYMPTOM AND SOCIAL‐COGNITIVE VULNERABILITY ASSOCIATIONS

Transdiagnostic research inherently involves examining symptoms spanning traditional diagnoses, but many transdiagnostic studies still often focus on symptoms that would be classified within one or two HiTOP spectra (e.g., internalizing and/or internalizing and disinhibited externalizing). Adopting assessment approaches spanning multiple HiTOP spectra, and multiple SCVs when possible, permits more compelling evaluations of the comparative degree of association for SCVs with specific HiTOP spectra, which is necessary for comprehensively understanding how HiTOP dimensions connect with treatment mechanisms. Multiple special issue papers demonstrate the value of these aspects of study design. Yang et al. (2026; this special issue) explicated associations for future time perspective, a construct involving how individuals conceptualize and evaluate their futures, with a broad range of symptom dimensions (e.g., thought disorder, internalizing) in a young adult sample. Their results inform hypothesis generation for future research testing mechanisms: For example, anticipation of negative consequences is implicated in internalizing development, but conversely, the development of disinhibited externalizing tendencies may be due to minimizing possible negative consequences. Additionally, Lloyd et al. (2026; this special issue) assessed a range of transdiagnostic dimensions and identified socio‐affective profiles guiding transdiagnostic intervention efforts.

Authors of both articles clearly connect their findings with treatment implications, and mapping HiTOP‐SCV associations can build on efforts such as these to improve the HiTOP's clinical translation and utility. Clinical research increasingly focuses on personalized diagnosis and treatment, and a more complete mapping of HiTOP‐SCV associations can aid clinicians in identifying which specific intervention strategies may be most likely to benefit individuals based on their dimensional assessment profiles. As an example, experiential avoidance and internalizing symptom associations are well‐documented. Research building on this knowledge could more fully elucidate the degree to which specific experiential avoidance facets are associated with other spectra in addition to internalizing across development, while also evaluating the degree to which other spectra are related to treatment outcome. For instance, behavioral avoidance and intolerance of social distress could potentially play an important role in the etiology of HiTOP detachment and anhedonia‐related symptoms (e.g., if reinforcing social situations are avoided over time). Assessing detachment in treatment studies would enable researchers to test the degree to which a range of different detachment‐based symptoms, versus symptoms of specific categorical diagnoses, change with treatment, and detachment assessment aligns well with novel transdiagnostic treatments targeting low positive affect and co‐occurring presenting issues (e.g., substance use, suicidality; Macrynikola et al., 2026). Furthermore, standing on other HiTOP spectra (e.g., antagonistic and disinhibited externalizing) may influence how detachment‐related issues manifest, and they could inform identification of cases wherein additional supports may be necessary to successfully plan and execute socially oriented exposure or behavioral activation activities.

We recognize that broadband assessment will be more realistic in some cases than others in research and practice (e.g., in some cases, lengthy assessments could overburden youth or parents/caregivers and delay treatment initiation). We also see time and resource‐related challenges as key opportunities for HiTOP‐informed assessment, which potentially offers efficiency advantages compared to traditional methods. For example, when using assessment approaches based on traditional categories, clinicians are often required to repeatedly assess the same general symptoms (e.g., insomnia, irritability) across many diagnostic modules. Assessment informed by the HiTOP potentially could streamline these efforts if the frequency and severity of symptoms repeated across traditional diagnoses can instead be assessed only once as part of a single HiTOP spectrum. Optimizing the efficiency of assessment will require intentionality when developing and validating new measures targeting HiTOP and SCV constructs, however, and we describe strategies for ensuring that administration feasibility and efficiency are priorities next.

## BRIDGING TRANSDIAGNOSTIC LITERATURES TO DEVELOP EFFICIENT MEASURES

When developing measures for use in broadband transdiagnostic studies, we encourage researchers to (a) precisely identify construct definitions and possible discriminant validity issues a priori and (b) strive to develop efficient measures that more easily permit assessment of multiple SCVs and symptom spectra. Regarding discriminant validity and “jingle‐jangle” issues, many SCV measures may assess general distress to a considerable extent (Gámez et al., 2011), which complicates interpretations when examining SCV‐symptom associations. Next, we imagine that researchers would prefer to assess a range of symptom dimensions and SCVs when possible, but that time and resource constraints often preclude doing so. We therefore encourage researchers to develop measures that are as efficient as possible toward aiding these goals, as many extant dimensional measures are lengthy and require significant administration times. Practical, but underutilized, strategies for efficient measure development include integrating Rasch‐ or other IRT‐based analytic strategies to optimize item sets, and open‐source data analytic vignettes and software packages are available to guide use of these tools (e.g., the easyRasch package; Johansson, 2026).

Collaborative research efforts that include authors with different areas of expertise may also augment transdiagnostic measure development efforts. For example, researchers with expertise in SCVs and treatment may benefit from collaboration with HiTOP researchers, who often have significant training in psychometrics and theories of measure development. In turn, HiTOP researchers may benefit from SCV and treatment researchers' perspectives by ensuring that measures assess aspects of thought, feeling, and behavior in a manner optimally informing treatment planning (we also recognize that these expertise summaries are oversimplifications and that many researchers have interests spanning these areas).

## CHALLENGES AND OPPORTUNITIES FOR CONSTRUCT DESCRIPTION IN TRANSDIAGNOSTIC RESEARCH

Optimizing terminology and construct descriptions used in HiTOP and SCV research may also improve their perceived clinical utility and clinical implementation, thereby enhancing the study design and measurement‐focused strategies discussed. Conversely, terminology that is unclear to clinicians and/or individuals accessing treatment may hinder clinical translation, which has been an issue raised in dimensional research already (e.g., for externalizing and antagonism description). Research focused on terminology and clarity of communication could evaluate how clinicians' perceptions of various descriptors connect with their treatment planning approaches (e.g., do some externalizing terms bring to mind different strategies than others). Additionally, qualitative research on lived experience often focuses on a single diagnosis (e.g., treatment experiences for individuals meeting criteria for one diagnosis), which has considerable value for understanding the impact of a diagnosis and related treatment experiences. However, due to the amorphous and co‐occurring nature of traditional diagnoses, clinicians, individuals accessing treatment, and their social supports often will discuss more than a single diagnosis throughout treatment, such that evaluating perceptions of transdiagnostic dimensions (e.g., emotion regulation) may provide a complementary perspective aligning with many individuals' real‐world experiences (Bellato & Seker, 2025). Lastly, related to special issue themes, research focused on clinical utility and perception of transdiagnostic terminology has often failed to consider the degree to which various descriptors apply across the lifespan. For example, assessment and treatment strategies focused on externalizing and interpersonal issues with youth often involve caregiver and parent‐mediated interventions, such that the perceived and actual utility of descriptors could vary meaningfully in these settings from adult‐focused intervention settings that often focus on individual therapy.

In closing, we also would like to acknowledge that many of our examples focused on connecting the HiTOP and SCVs would be relevant to other mechanisms as well (e.g., mechanisms from the National Institute of Mental Health Research Domain Criteria), and other research teams have identified many of the core issues reviewed. We hope that synergistically discussing these issues here provides researchers with ideas for sharpening the joint assessment of dimensional symptoms and SCVs, toward maximizing the potential of both frameworks in research and clinical practice.

## AUTHOR CONTRIBUTIONS

**Kasey Stanton:** Conceptualization (lead); writing—original draft (lead); review and editing (lead). **Jack D. Burke:** Conceptualization (supporting); writing—review and editing (supporting). **Helena Towne:** Writing—review and editing (supporting). **Isabella Whitesides:** Writing—review and editing (supporting).

## CONFLICT OF INTEREST STATEMENT

The authors declare no conflicts of interest.

## ETHICAL CONSIDERATIONS

Ethics requirements do not apply, as no data are presented nor was any new research conducted for this editorial perspective article.

## Data Availability

Data sharing not applicable to this article as no datasets were generated or analyzed for this editorial perspective article.
